# Spectroscopic and Theoretical Studies of Fluorescence Effects in 2-Methylamino-5-(2,4-dihydroxyphenyl)-1,3,4-thiadiazole Induced by Molecular Aggregation

**DOI:** 10.1007/s10895-017-2175-2

**Published:** 2017-09-09

**Authors:** Arkadiusz Matwijczuk, Andrzej Górecki, Marcin Makowski, Katarzyna Pustuła, Alicja Skrzypek, Joanna Waś, Andrzej Niewiadomy, Mariusz Gagoś

**Affiliations:** 1 Department of Biophysics, University of Life Sciences in Lublin, Akademicka 13, 20-950 Lublin, Poland; 2 Department of Physical Biochemistry, Faculty of Biochemistry, Biophysics and Biotechnology, Jagiellonian University, Gronostajowa 7, 30-387 Krakow, Poland; 3 Department of Theoretical Chemistry, Faculty of Chemistry, Jagiellonian University, Ingardena 3, 30-060 Kraków, Poland; 4 Department of Chemistry, University of Life Sciences in Lublin, Akademicka 15, 20-950 Lublin, Poland; 5 Departament of Chemistry, Jagiellonian University, Ingardena 3, 30-060 Kraków, Poland; 6 Institute of Industrial Organic Chemistry, Annopol 6, 03-236 Warsaw, Poland; 7 Department of Cell Biology, Institute of Biology, Maria Curie-Skłodowska University, Akademicka 19, 20-033 Lublin, Poland

**Keywords:** Molecular spectroscopy, 1,3,4-Thiadiazole, Molecular aggregation, DFT calculations, The effect of two fluorescence emission bands

## Abstract

The article presents the results of fluorescence analyses of 2-methylamino-5-(2,4-dihydroxyphenyl)-1,3,4-thiadiazole (MDFT) in an aqueous environment. MDFT dissolved in aqueous solutions with a pH value in the range from 1 to 4.5 yielded an interesting effect of two clearly separated fluorescence emissions. In turn, a single fluorescence was observed in MDFT dissolved in water solutions with a pH value from 4.5 to 12. As it was suggested in the previous investigations of other 1,3,4-thiadiazole compounds, these effects may be associated with conformational changes in the structure of the analysed molecule accompanied by aggregation effects. Crystallographic data showed that the effect of the two separated fluorescence emissions occurred in a conformation with the –OH group in the resorcyl ring bound on the side of the sulphur atom from the 1,3,4-thiadiazole ring. The hypothesis of aggregation as the mechanism involved in the change in the spectral properties at low pH is supported by the results of (Time-Dependent) Density Functional Theory calculations. The possibility of rapid analysis of conformational changes with the fluorescence spectroscopy technique may be rather important outcome obtained from the spectroscopic studies presented in this article. Additionally, the presented results seem to be highly important as they can be easily observed in solutions and biologically important samples.

## Introduction

One of the major goals of modern medicine is to address the rapidly increasing problem of cancer diseases. It is estimated that neoplasm diseases will affect one in four inhabitants of highly developed countries in the near future. The most important clinical problem associated with the use of chemotherapeutic agents in cancer therapy is the high toxicity of anti tumour drugs which are already available or are being developed. The most effective compounds offering the greatest hope are represented by 1,3,4-thiadiazoles with a substituted resorcyl fragment. The thiadiazol group is a system with comprehensive biological activity [1, 2]. The activity of 1,3,4-thiadiazoles is related to the presence of a thioimine group in their structure. As indicated in papers by Siddiqui and collaborators [3], this compound group has been investigated worldwide more extensively than all the other thiadiazol isomers. Investigations of 1,3,4-thiadiazole compounds, in particular their di-substituted derivatives, represent a majority in the available literature, probably due to their pharmacological effects. Literature presents the entire thiadiazol family as compounds with anticancer [2, 4–6], antifungal [7], antibacterial [7], anticonvulsant [8], anti-inflammatory [9, 10], antihypertensive [9], antiviral [7], radioprotective [7], antidepressant and antioxidant [11], or insecticidal [9] activity.

**Fig. 1:**
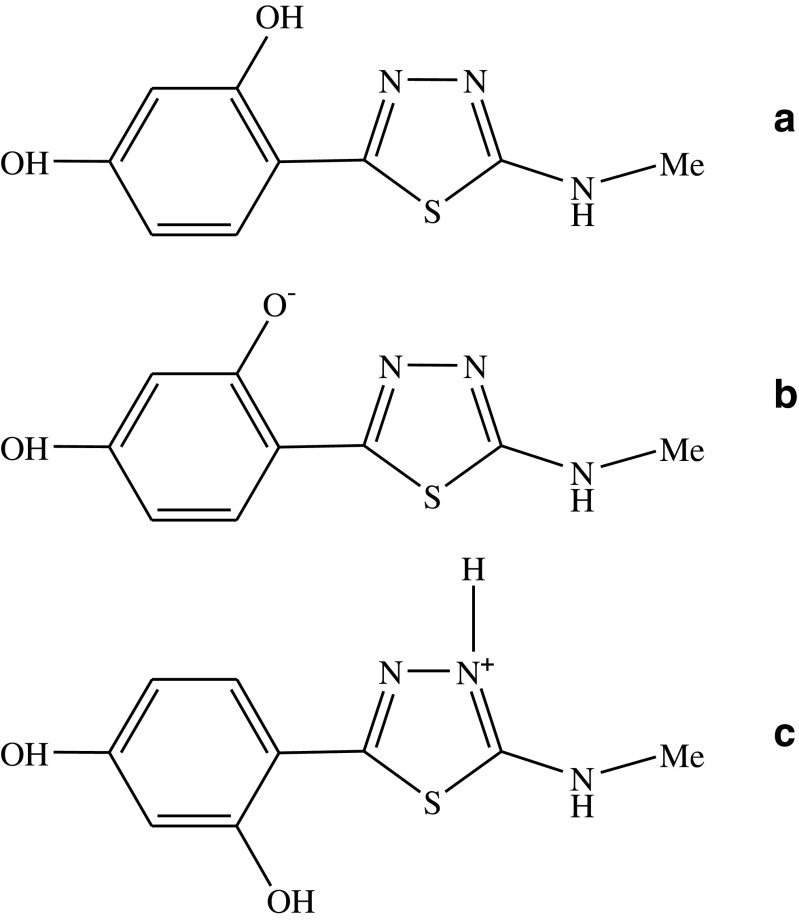
Chemical structure of the MDFT molecule (**a** enol form, **b** form ionised with the –O^−^ group, **c** form ionised with the–N^+^–H group, in which the–OH group is located on the side of sulphur from the 1,3,4-thiadiazole ring)

Given its confirmed and promising therapeutic activity, 2-methylamino-5-(2,4-dihydroxyphenyl)-1,3,4-thiadiazole (MDFT, Fig. 1a) was chosen for the investigations of the mechanism of molecular interactions presented in this paper [10]. Additionally, the 1,3,4-thiadiazol compounds chosen for the study exhibit very interesting effects related to their pharmacological and spectroscopic applications, e.g. the effects of keto/enol tautomerism induced by changes in environment polarizability [12–15], crystal polymorphism effects [16], solvatomorphism [17], and the very interesting interactions in model lipid systems [18, 19]. The selected test compounds of group 1,3,4-thiadiazols are also very interesting group of ligands, forming complexes with metal ions block d [20]. The presented 1,3,4-thiadiazols mainly exhibit interesting dual fluorescence [21–23] or two separate emissions, but the mechanism of these effects have not been fully recognised and elucidated despite the extensive research. The combination of the spectroscopic and structural effects presented in the aforementioned papers is extremely important for elucidation of the pharmacological phenomenon of the analysed 1,3,4-thiadiazols.

The main aim of this study was to provide spectroscopic analyses of the molecular organisation of MDFT in an water medium at different concentrations of hydrogen ions and temperature fluctuations. With the use of spectroscopic methods, e.g. electronic absorption spectroscopy, fluorescence technique with RLS, and measurements of fluorescence lifetimes, the complexity of the physical processes involved in the MDFT fluorescence effects induced by changes in the hydrogen ion concentration was demonstrated. Based on the fluorescence measurements and the accessibility X-ray crystallography data, an attempt at experimental and theoretical elucidation of these effects were made. As indicated by the crystallographic studies, the rotation of the resorcyl fragment of the molecule and the site of hydrogen atom protonation, depending on the pH of the environment of crystal growth, yield the fluorescence effects which were mentioned above.

The effects associated with dual fluorescence [24–33] or two clearly shifted fluorescence emissions are related to emergence of two distinctly separated emission spectra induced by electronic excitation. They are usually associated with the solvent polarity, pH of the environment, temperature, and molecular aggregation. The most common attempts at elucidation of these effects take into account processes connected with the appearance of intra molecular CT states [34–39] or CT states with TICT (Twisted Intramolecular Charge Transfer) [40–50]. Another equally interesting and very popular explanation of the phenomena mentioned above is the Excited-State Intra molecular Proton Transfer (ESIPT) process [25, 51–56]. Such effects are usually observed in organic molecules in which the acceptor group is located close to the proton-donor group. Similar effects can also be explained by the existence of excimer fluorescence [57–64]. In addition, it is worth to mention announced by Brancato and associates a result “anti - Kasha”, by which we are also seeing the effect of dual fluorescence [65].

The fluorescence spectroscopy analyses of MDFT in a aqueous environment revealed an effect of two distinctly separated and largely overlapping fluorescence emissions. This effect can be induced by the hydrogen ion concentration, temperature fluctuations, solvent polarity, and aggregation effects. Detailed analysis of the organisation of the investigated molecules in an aqueous environment is important for understanding the pharmacological applications of 1,3,4-thiadiazoles.

## Materials and Methods

### Materials

The 2-methylamino-5-(2,4-dihydroxyphenyl)-1,3,4-thiadiazole (MDFT) compound (see Fig. 1) was synthesized in the Department of Chemistry of the University of Life Sciences in Lublin; details of the procedure are described elsewhere [10].

The purification procedure of the MDFT compound is described in detail in references [16, 17, 66].

### Methods

All solutions were measured with an Elmetron CP-502 pH-meter at room temperature. For methanol-MDFT solutions, MDFT was first dissolved in methanol and the pH was changed by slow addition of 0.1M HCl to the glass flask. For water-MDFT solutions, 0.1M NaOH was first added to water to obtain pH 12. Next, powdered MDFT was dissolved in water. To obtain the specified pH of the water-MDFT solution, 0.1M HCl was slowly added. The pH was continually controlled.

#### Electronic Absorption and Fluorescence Spectra

Electronic absorption spectra of MDFT were recorded on a double-beam UV–Vis spectrophotometer Cary 300 Bio (Varian) equipped with a thermostatted tray holder with a 6 × 6 multi-cell Peltier block. The temperature was controlled with a thermocouple probe (Cary Series II from Varian) placed directly in the sample.

Fluorescence excitation, emission, and synchronous spectra were recorded with a Cary Eclipse spectrofluorometer (Varian) at 22 °C. Fluorescence spectra were recorded with 0.5 nm resolution and corrected for the lamp and photomultiplier spectral characteristics. Resonance light scattering (RLS) measurements were performed as shown in Pasternack and Collings [67]. The excitation and emission monochromators of the spectrofluorimeter were scanned synchronously (0.0 nm interval between excitation and emission wavelengths); the slits were set to obtain spectral resolution of 1.5 nm. The spectral analysis was performed with the use of Grams/AI 8.0 software (Thermo Electron Corporation).

#### Time-Correlated Single Photon Counting (TCSPC)

Time-correlated single photon counting (TCSPC) measurements were performed using a FluoroCube fluorimeter (Horiba, France). The samples were excited with pulsed Nano- LED diode at 372 nm (pulse duration of 150 ps) operated with 1 MHz repetition. To avoid pulse pile-up, the power of the pulses was adjusted to an appropriate level using a neutral gradient filter. Fluorescence emission was recorded using a picosecond detector TBX-04 (IBH, JobinYvon, UK). The Data Station and DAS6 software (JobinYvon (IBH, UK)) were used for data acquisition and signal analysis. All fluorescence decays were measured in a 10 × 10 mm quartz tray using an emitter bandpass filter with a centre wavelength of 420 and 40 nm bandwidth. The excitation profiles required for the simplified analysis were measured without the emitter filters on a light scattering tray. All measurements were performed in water at 20 °C and various pH values. Each case of fluorescence decay was analysed with a multi-exponential model shown in the equation:

1 $${I_t}=\sum\limits_{i} {{\alpha _i}\exp \left( {{\raise0.7ex\hbox{${ - t}$} \!\mathord{\left/ {\vphantom {{ - t} {{\tau _i}}}}\right.\kern-0pt}\!\lower0.7ex\hbox{${{\tau _i}}$}}} \right)}$$

where α_i_ and τ_i_ are the pre-exponential factor and the decay time of component *i*, respectively.

The best-fitted parameters were obtained by minimization of the reduced χ^2^ value as well as residual distribution of the experimental data. The fractional contribution (*f*_*i*_) of each decay time and the average lifetime of fluorescence decay (<τ>) were calculated with the following equations:

2 $${f_i}=\frac{{{\alpha _i}{\tau _i}}}{{\sum\nolimits_{j} {{\alpha _j}{\tau _j}} }}$$

3 $$\left\langle \tau \right\rangle =\sum\nolimits_{i} {{f_i}{\tau _i}}$$

### DFT Calculations

The DFT calculations were performed with the Gaussian 09 package [68] using B3LYP exchange–correlation functional [69] and 6-31G** basis set [70]. The dispersion effects were accounted for in the framework of Grimme’s D3 model augmented by the Becke-Johnson damping scheme [71]. The solvent effects were modelled by PCM formalism [72]. The excited state treatment was carried out using a standard RPA approach to TD DFT formalism [73] while the influence of the solvent on energetics was determined according to the linear response approximation.

## Results and Discussion

### Spectroscopic Studies of the MDFT Fluorescence Effects in Aqueous Solutions

**Fig. 2:**
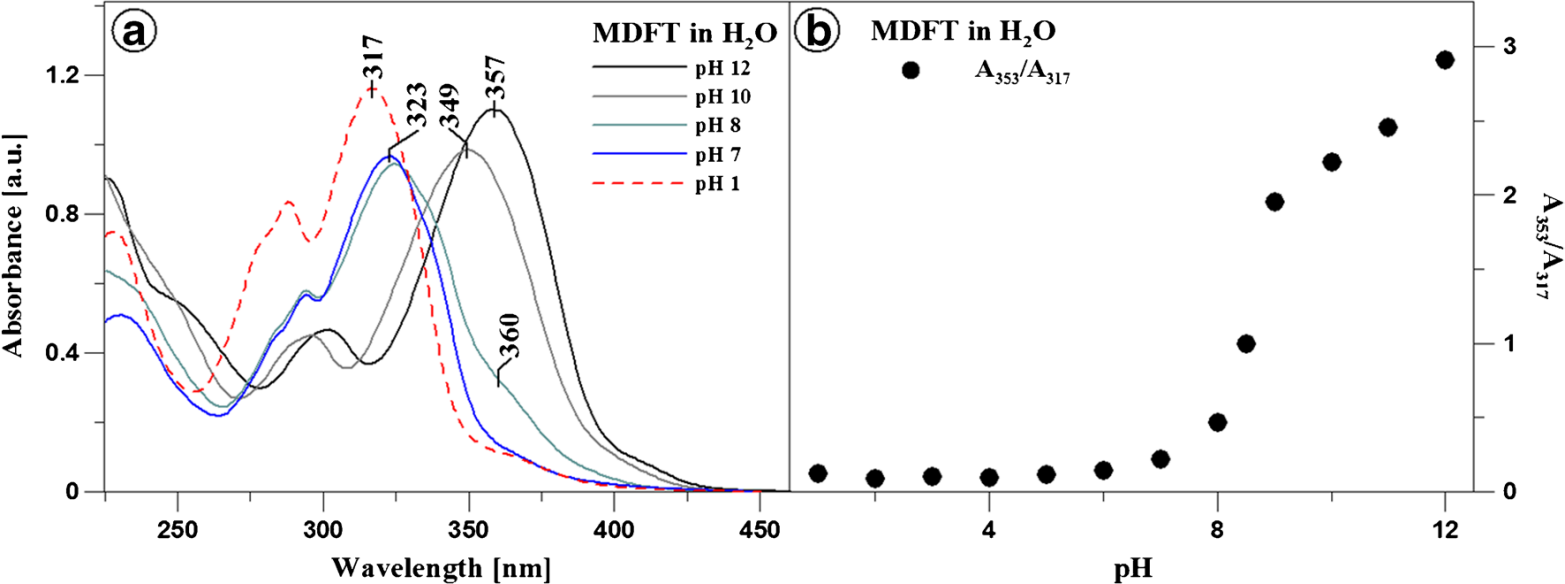
MDFT electron absorption spectra obtained in the aqueous solution at pH 12, 10, 8, 7, 1 (Panel **a**). Panel **b** in the inset presents the ratio of the 353 to 317 absorption maximum depending on the changes of the pH of the environment

Analyses of the MDFT performed in the aqueous solution over the entire pH range indicate distinct changes in the positions of absorption bands, especially in the region that is important for physiological values. Figure 2a presents the electron absorption spectra of MDFT obtained at various pH values (pH 1, 7, 8, 10, and 12, respectively). Dissociation of the –OH group of the resorcyl ring in the orto position (Fig. 1b) causes a hypsochromic spectral shift (by 2949 cm^− 1^) in the case of the MDFT spectrum at pH 12 and a bathochromic shift (by 586 cm^− 1^) for the spectrum at pH 7. The ionisation process can also be accompanied by processes of compound aggregation [74]. The increase in the MDFT absorption bandwidth at pH 7.5–8 suggests possible presence of other than monomeric spectral forms of the compound [75]. The compound absorbance for the spectrum at pH 2 has the lowest value, which indicates predominance of the aggregated forms. Unexpectedly, in the case of the MDFT spectrum at pH 1, absorbance significantly increased, which may suggest processes associated with monomerisation of the compound. Panel b in Fig. 2 shows the ratio of the maximum absorbance at 353 nm (predominant monomeric form) to that at 317 nm (predominant associated form), depending on the pH of the aqueous solution. As can be seen, the MDFT monomerisation level (i.e. the predominance of the monomeric form) is the highest at pH 12 and exhibits a minimum value at pH 1. The greatest changes in the presented ratio are especially evident at pH 6–8, which is associated with the pK point associated with protonation of the nitrogen atom (located closer to the NH group in the substituent group) from the 1,3,4-thiadiazol ring.

**Fig. 3:**
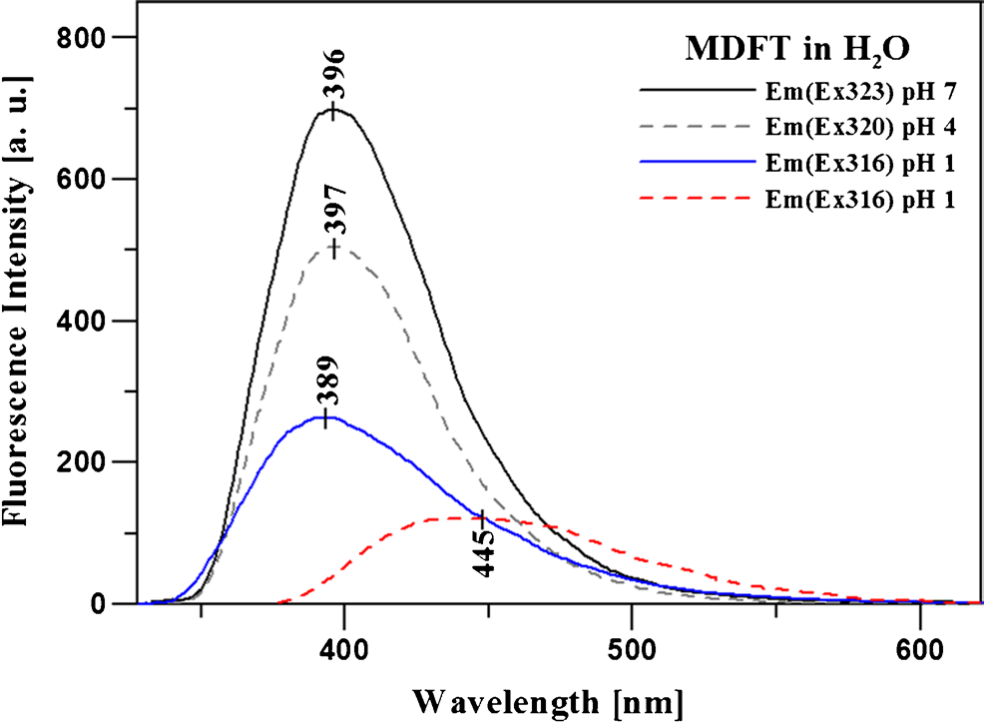
MDFT fluorescence emission spectra obtained in the aqueous solution at various pH values (pH 1, 4, and 7, respectively). The Em(Ex323) symbol denotes emission at an excitation wavelength of 323 nm

The fluorescence spectroscopy measurements were carried out in the subsequent stage of the study of the spectroscopic properties of MDFT. Together with the change in the pH of the solution, there was a single fluorescence effect or two, clearly separated, partially overlapping emission spectra. Given the analogy to crystals growing in an acidic environment and suggesting a molecule conformation associated with the observed effect, analyses in aqueous solutions were performed for better elucidation of the effect demonstrated by MDFT. In Fig. 3, the effect of two clearly separated and overlapping fluorescence emissions can be observed in the pH range of 1–3.5 and single fluorescence is visible for pH 4–12. In the other polar and non-polar solvents, only single fluorescence is visible (not shown). Figure 3 presents selected spectra of MDFT fluorescence emission obtained at different pH values in the aqueous solution (pH 1, 4, and 6, respectively). The excitation wavelength for all the analysed samples was 323 nm at pH 6, 320 nm at pH 4, and 316 nm and 360 nm at pH 1. As shown, single fluorescence with a maximum at ca. 400 nm (for both excitations) can be observed at pH 6 (and up to pH 12). At pH 4, an increase in the bandwidth and a slight bathochromic shift of the emission spectrum are visible. Next, the decrease in the pH values (between pH 3.5 and 1) is accompanied by additional fluorescence with a maximum at 450 nm at an excitation wavelength of 360 nm. Moreover, unlike in the case of the dissolved MDFT spectra, a ca. 100-nm short-wave shift of the emission spectra is observed in the solid form (not shown) [21]. This effect can be attributed to the denser arrangement of MDFT molecules in the crystal than in the environment of various solutions. Additionally, in the case of the fluorescence emission spectra for excitation with a 360-nm wavelength, a significant increase in the bandwidth and a decrease in the band intensity can clearly be seen. The inset in Fig. 3 presents the structure of the MDFT molecule crystallized in H_2_O [66]. Furthermore, it can be noted that the ratio of the MDFT fluorescence maxima over a wavelength range from 396 to 450 nm depending on the pH of the solutions changing from pH 1 to pH 3.5 almost does not change. This implies that the process of protonation of the nitrogen atom in the heterocyclic ring (Fig. 1) is in equilibrium with the enol form of the compound and does not change the ratio. Above pH 3.5, the ratio increases substantially and the fluorescence with a maximum at ca. 450 nm almost disappears.

**Fig. 4:**
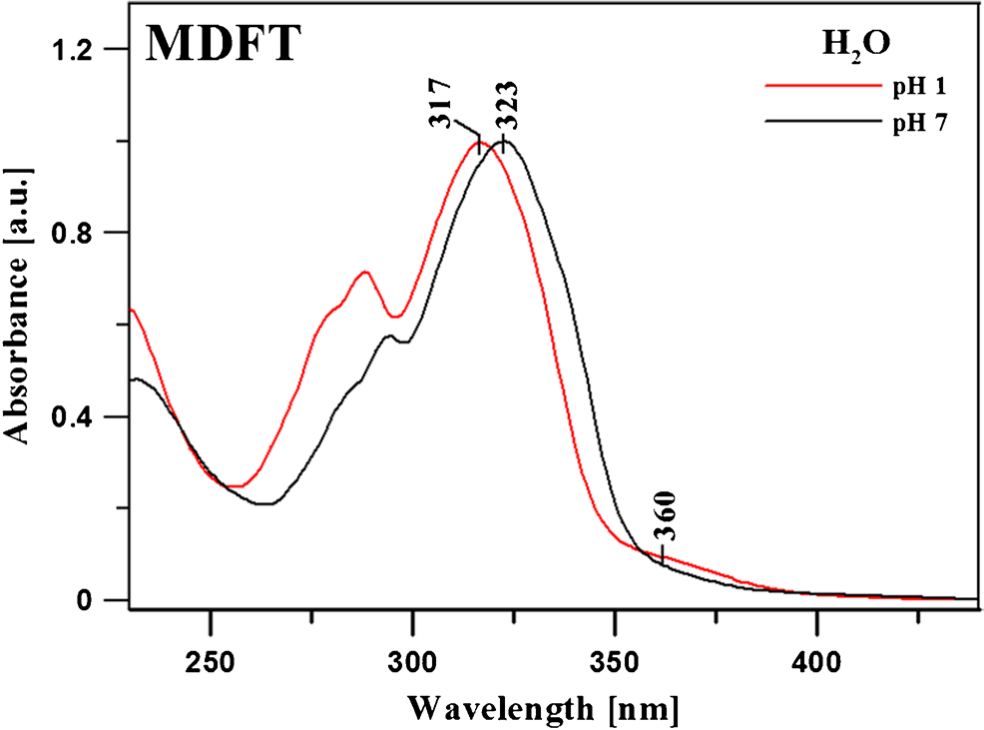
Normalised MDFT electron absorption spectra obtained in the aqueous solution at pH 7 and 1 (red line and black dashed line, respectively)

Figure 4 shows electron absorption spectra for MDFT in the aqueous solution with pH 6 (black line) and in the same solution acidified with 0,1 M HCl to pH 1 (red line). Both spectra were normalised at a maximum wavelength for easier interpretation. As can be noted, there are evident concurrent bathochromic and hypsochromic shifts. For MDFT dissolved in the aqueous solution, a bathochromic shift from 323 nm (30,960 cm^− 1^) at pH 7 to 360 nm (27,778 cm^− 1^) at pH 1 and a hypsochromic shift to 317 nm (31,546 cm^− 1^) at pH 1 are visible (Δν between 323 nm and 360 nm is 3182 cm^− 1^). This characteristic effect involves molecular aggregation processes [76]. The Resonance Light Scattering RLS technique (described below) and DFT quantum–mechanical calculations (described below) most probably indicate MDFT chromophore aggregation effects [21]. Based on the exciton splitting theory and the spectral shifts, it was possible to calculate the distance between the adjacent chromophores of the MDFT molecules [67]. The distance between the adjacent chromophores R_β_ can be calculated from equation:

4 $${R_\beta }=1.71\;\sqrt[3]{{\frac{{{\mu ^2}\kappa }}{{{\eta ^2}\beta }}}}$$

where µ is the transition dipole moment of interacting molecules, η – refractive index, β – energy of the dipole–dipole interaction (calculated in a classical way). The exciton model takes into account an aggregated structure, formed by identical particles, in which transition dipole moments of adjacent molecules are parallel; hence, *α* = 0 (where *κ* = 1–3cos^2^θ, where θ is an angle between the transition dipole moments of the neighbouring molecules). *κ* = 1 for the *card pack* molecule aggregates and *κ* = − 2 for the *head to tail* molecular aggregation type. The transition dipole moment for the MDFT monomer calculated by integration of the absorption spectrum has a value of *µ* = 4.6 D (in H_2_O). In the case of MDFT dimers in the aqueous solution, the distance between the adjacent chromophores is estimated at 3.77 Å [67]. These results are consistent with crystallographic data, where the distance between adjacent molecules in the crystal growing in an aqueous solution was 3.35 Å [66]. This distance in crystals is obviously smaller than that calculated for the solutions due to the evidently denser arrangement of crystal molecules and the enhanced strength of inter-molecular interactions.

### TCSCP Study

**Fig. 5:**
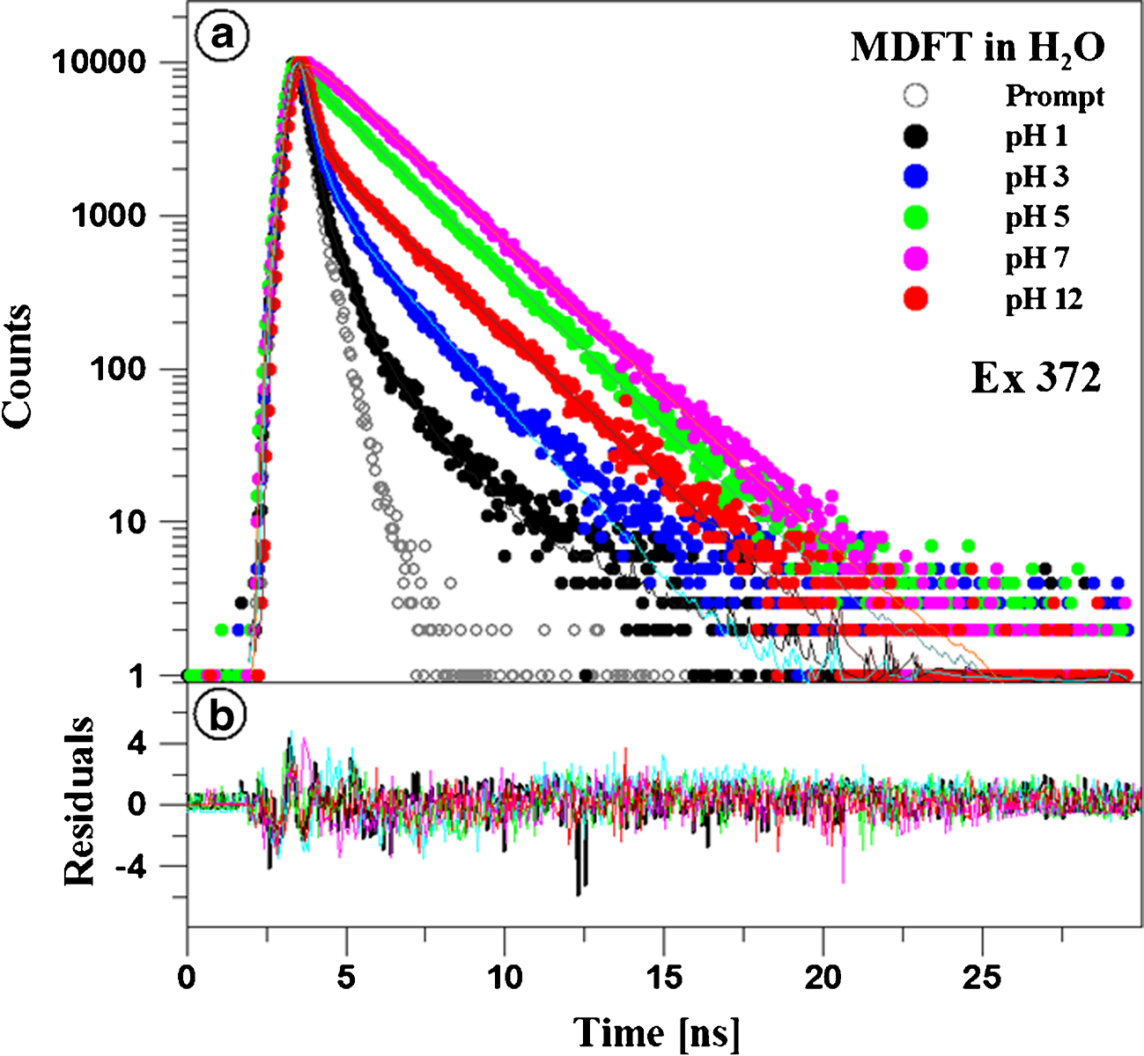
The effect of pH on the fluorescence decay in MDFT. In panel **a**, the dotted curves show the decay of MDFT fluorescence emission observed with the TCSPC technique in water at a specified pH value, and the solid lines are double exponential fits. Panel **b** shows the plots of residuals (determined for the data in panel **a**). The excitation pulse profile, set up at 372, is shown by the black dotted curve

**Fig. 6:**
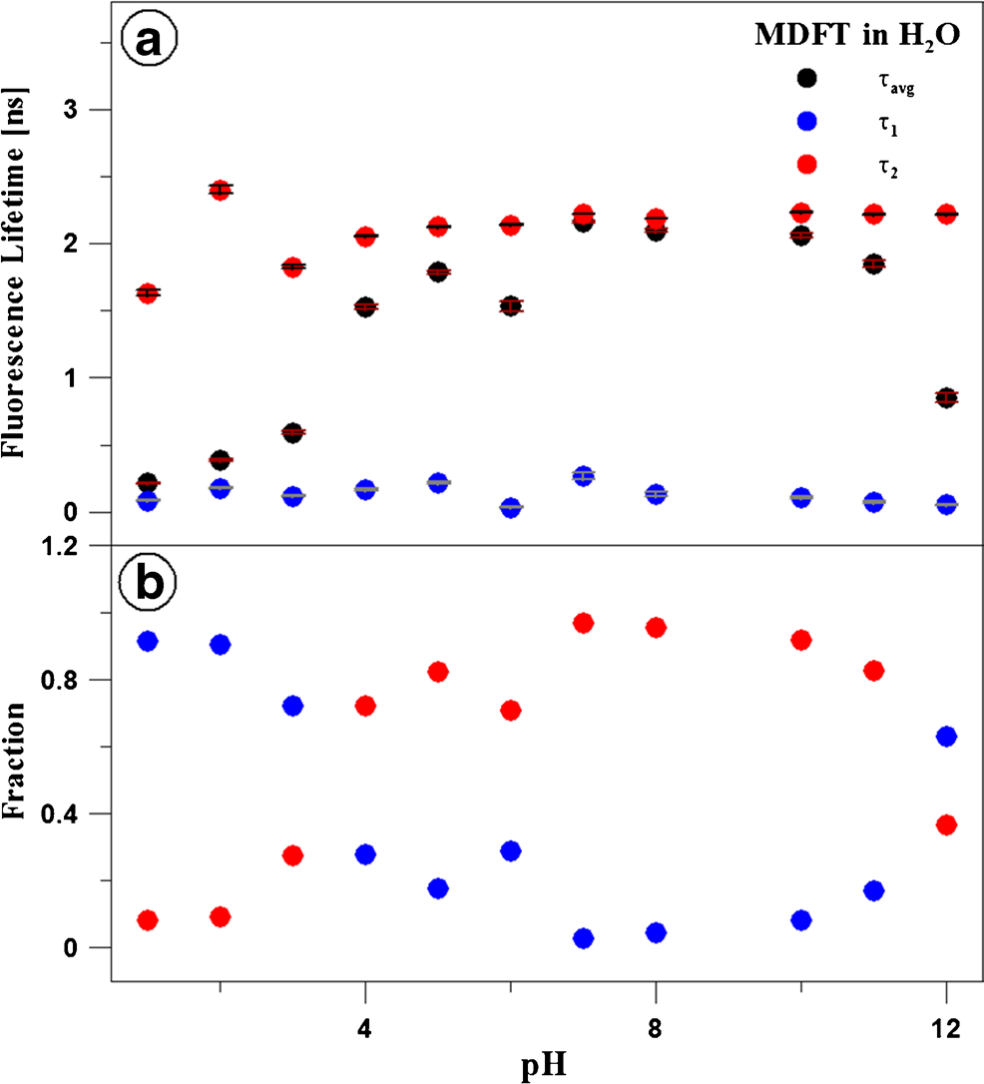
The effect of pH on the fluorescence lifetime of MDFT. Panel **a** presents fluorescence lifetimes of the first (τ_1_) and the second (τ_2_) component of MDFT, together with the mean values observed at various pH. The dependences of the fractional contribution of both components on pH are presented in panel **b**. The data analysis was carried out for a two-component model (Eq. 1 for *i* = 2) for data presented in Fig. 6

Figures 5 and 6 as well as Table 1 present the results of measurements of fluorescence lifetimes for MDFT in the aqueous solution over the entire pH range (shown for 1, 7, 8, and 12). The excitation wavelength (372 nm) is appropriate for the long-wave edge of absorption of the monomeric form and the resonant excitation of the aggregated form (~ 370 nm) at pH 7. In turn, at a high pH level, ionised MDFT forms, i.e. monomers, are excited (see Fig. 4), and aggregates are resonance-excited at low pH values. The fluorescence decay was monitored with the TCSPC method at 22 °C for a wavelength light range of 400–440 nm. The results were analysed by deconvolution of the fluorescence decay with the use of the apparatus profile and Eq. (1), each time for *i* = 1, 2, and 3. The two-exponential fluorescence decay model was optimal in all the cases. The mono-exponential decay was insufficient, and the inclusion of the third component did not improve the quality of the fit, which was verified by the value of the fit parameter and the residue distribution analysis (Fig. 5b). Both fluorescence lifetime components exhibit low variation over the entire pH range, see Fig. 6a. The shorter one, on average 0,13 ± 0,07 ns, can be considered constant over the analysed range. The fluorescence lifetime for the second component is 2.19 ± 0.04 ns for pH values higher than 5. For the more acidic solutions, a slightly reduced lifetime can be noted, i.e. 1.62 ± 0.02 ns for pH 1. Considerably greater variability is observed in the case of the fractional contribution of the components described. At pH 7, the longer-lifetime component is clearly predominant, as its fractional contribution reaches a value of 97%. In turn, the fractional contribution declines at both the lower and the higher pH values. In the case of acidic solutions, its fractional contribution drops to 8% (at pH 1), and the greatest change is noted between pH 4 and 3. In alkaline solutions, the lowest fractional contribution of the component was detected at pH 12 (37%), and the greatest decline was observed between pH 11 and 12. Since the analysis of the fluorescence intensity decay clearly indicates the presence of two components with substantially different lifetimes, these components can be identified with the different fluorophore forms. In this interpretation, the contribution of the observed components (fractal contribution) monitors the transition of the equilibrium between these forms together with changes in the solvent pH. This change is most pronounced in a pH range corresponding to the first pK of the analysed compound; therefore, a short lifetime can be regarded characteristic for the protonated/positively ionised form, while a shorter lifetime is typical of the enol form. Another change in the contribution of the fractions is evident in a pH range close to the second pK, which indicates that the shorter lifetime is characteristic for the deprotonated/negatively ionised MDFT form. Literature often presents analysis based on the value of the mean fluorescence lifetime; therefore, based on these components of fluorescence lifetimes and their fractal contribution, the mean fluorescence lifetimes specific to the different pH values were determined using Eq. (3). Given the low variability of the values of the fluorescence lifetime components, the relationship is highly similar in its course to the distribution of the contribution of the longer-lifetime component. Importantly, in a pH range in which single fluorescence is observed in the emission spectra, the lifetimes are substantially reduced to approx. 0.45 ns. In contrast, in the region exhibiting longwave fluorescence, the mean fluorescence lifetime increases considerably up to ca. 1.3 ns. The effect of the lengthening of the fluorescence lifetime in the case of molecules in which similar fluorescence phenomena are observed is characteristic for excimer interactions [57], in contrast to processes induced by the aggregation (dimerisation) phenomenon, which is usually characterised by clear reduction [77] or decay of the lifetime.

**Table 1:** Fluorescence lifetimes of MDFT in H_2_O in relation to changes in pH

MDFT in aqueous solution	
pH	τ±Δτ
1.0	0.21 ± 0.01
2.01	0.39 ± 0.01
3.0	0.59 ± 0.01
4.0	1.53 ± 0.02
5.01	1.79 ± 0.01
6.03	1.53 ± 0.04
7.01	2.16 ± 0.01
8.06	2.10 ± 0.01
9.0	2.10 ± 0.01
10	2.06 ± 0.02
11.02	1.85 ± 0.02
12	0.85 ± 0.03

### RLS Study

**Fig. 7:**
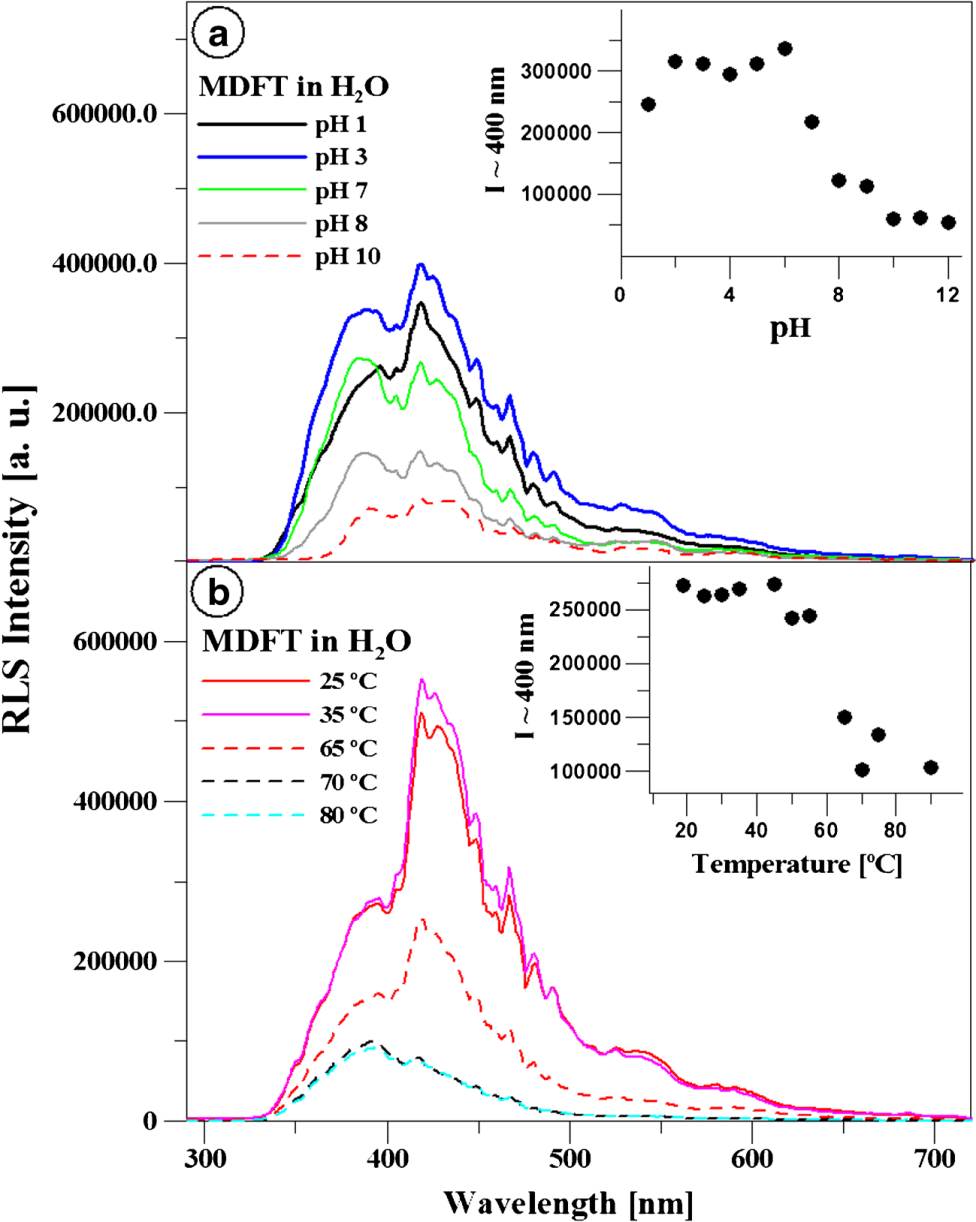
Panel **a** RLS (Resonance Light Scattering) spectra for MDFT obtained in the aqueous solution for the different pH values. The figure presents pH 1, 3, 7, 8, and 10, respectively. The inset in Panel **a** presents the ratio of the RLS signal intensity at a wavelength of 400 nm accompanying pH changes in the solution. Panel **b** RLS spectra for MDFT obtained in the aqueous solution at pH 3 and different temperatures (shown: temperature rise; not shown: temperature fall). The inset in Panel **b** presents the ratio of the RLS signal intensity at a wavelength of 400 nm accompanying the temperature fluctuations

In order to investigate the impact of aggregation on the presented fluorescence effects, RLS spectra for MDFT were obtained in water at fluctuations in temperature and pH of the environment. Panel a in Fig. 7 presents RLS (Resonance Light Scattering, Δλ = 0) spectra for MDFT generated in the aqueous solution at varying pH values. Panel b shows selected RLS spectra for MDFT obtained in the aqueous solution at pH 2.5 and temperature fluctuations. As indicated in e.g. Pasternak’s and Parkash’s investigations, the presence of the RLS bands should be attributed to chromophore aggregation of the components contained in the solution [67, 78]. As can be seen, RLS bands with greater or lesser intensity are present in water at different temperature and pH values. It is evident that the increase in pH is accompanied by a decline in the RLS signal. Panel a in the inset in Fig. 7 presents a decline in the intensity of the RLS signal accompanying the increase in the pH value. The RLS signal loses its intensity substantially at a pH value close to 4. In Panel b, a decline in the RLS signal accompanying the temperature rise is clearly visible, and the inset shows a sharp loss of the signal at a temperature of ca. 60 °C. Above this temperature, the two clearly separated fluorescence emissions disappear as well. RLS spectra and their dependence on the environmental pH and temperature clearly support the correlation of the presented effect with molecular aggregation. The oscillatory structure of the RLS bands indicates many aggregated MDFT structures, which can vary in size. The observed effect is fully reversible in this case (not shown for clarity of the presented results).

### Ex-(1-T)

**Fig. 8:**
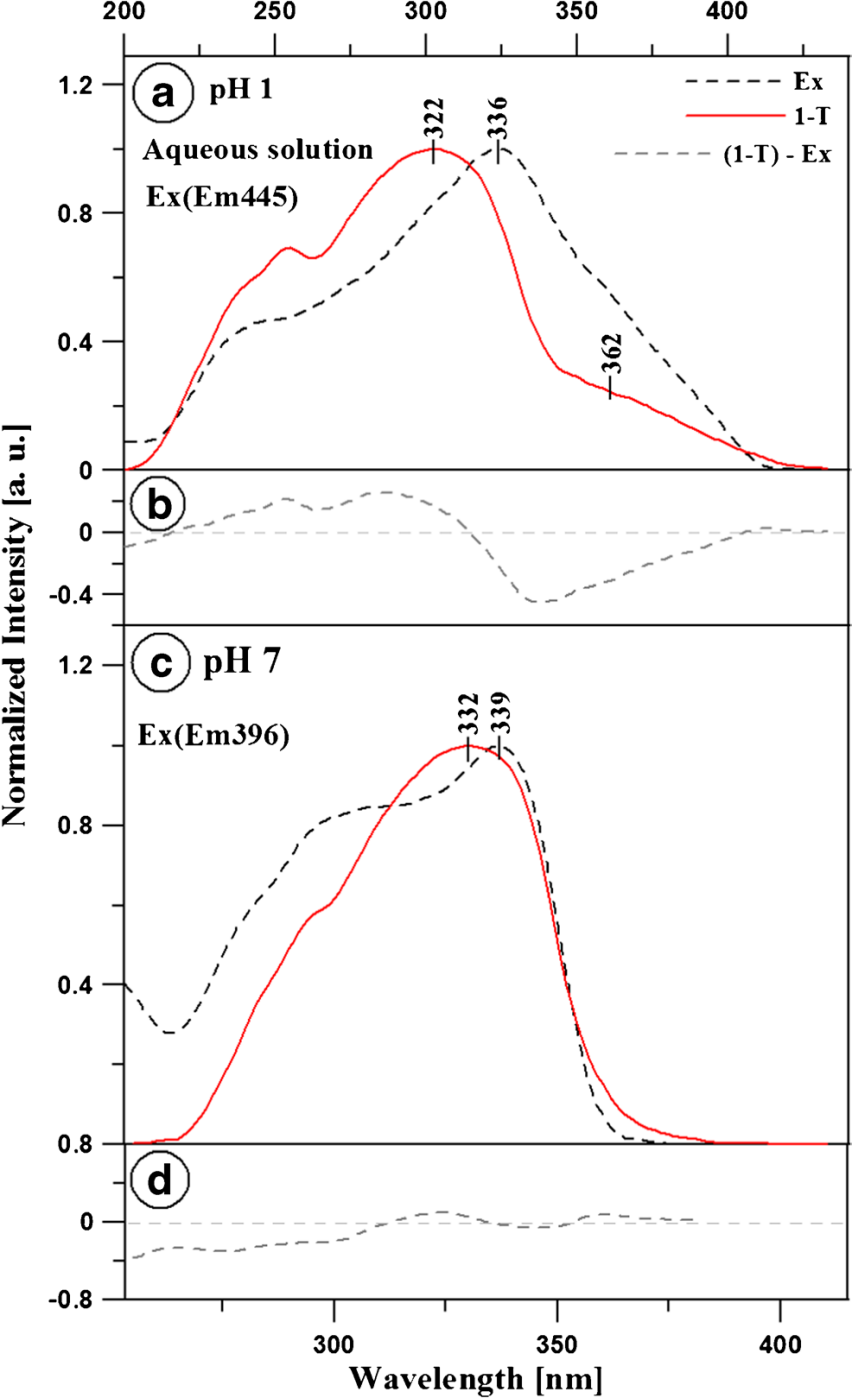
Panels **a** and **b** show fluorescence excitation spectra for MDFT in H_2_O at pH 1 (Panel **a**) and at pH 7 (Panel **b**) in comparison with the 1-T (T – Transmission) spectra. The excitation wavelength at pH 1 is 445 nm (black lines) and 396 nm (red lines)

In subsequent analysis of the MDFT dual fluorescence and the association of the observed effects with the phenomenon of chromophore aggregation in the compound shown in Fig. 8, the MDFT fluorescence excitation spectra obtained in the aqueous solution at pH 1 and 7 (Panels a and c, respectively) were presented for the same samples in comparison with the 1-T spectra (T – transmission). In turn, Panels b and d show differential spectra (1–T)–(Ex). The excitation emission was observed at a wavelength of 445 nm (pH 1) and 396 nm (pH 6), respectively. In the case of the fluorescence excitation spectra, in comparison with the 1-T spectra for both samples at pH 1, distinct bands on the long wave side of the spectrum can be observed, which should be associated with the aggregated form of excited MDFT molecules. Panel a shows that the 1-T MDFT spectrum at pH 1 is hypsochromically shifted, whereas a clear bathochromic shift is visible at pH 7 (Panel c). In accordance with the exciton splitting theory, these shifts are related to two types of aggregation: the hypsochromic shift is associated with “card pack” aggregates and the bathochromic shift is attributed to “head to tail” aggregation [79]. In an alkaline environment, in which the compound is monomerised, a substantially lower number of bands from aggregated forms can be observed. Therefore, a conclusion can be drawn that the effect of two separate fluorescence emissions in the MDFT molecule is clearly associated with the effect of molecular aggregation of the compound in the aqueous solution. The RLS analyses which are presented in this article largely confirm this assumption.

### Temperature Effects in MDFT Fluorescence Studies

**Fig. 9:**
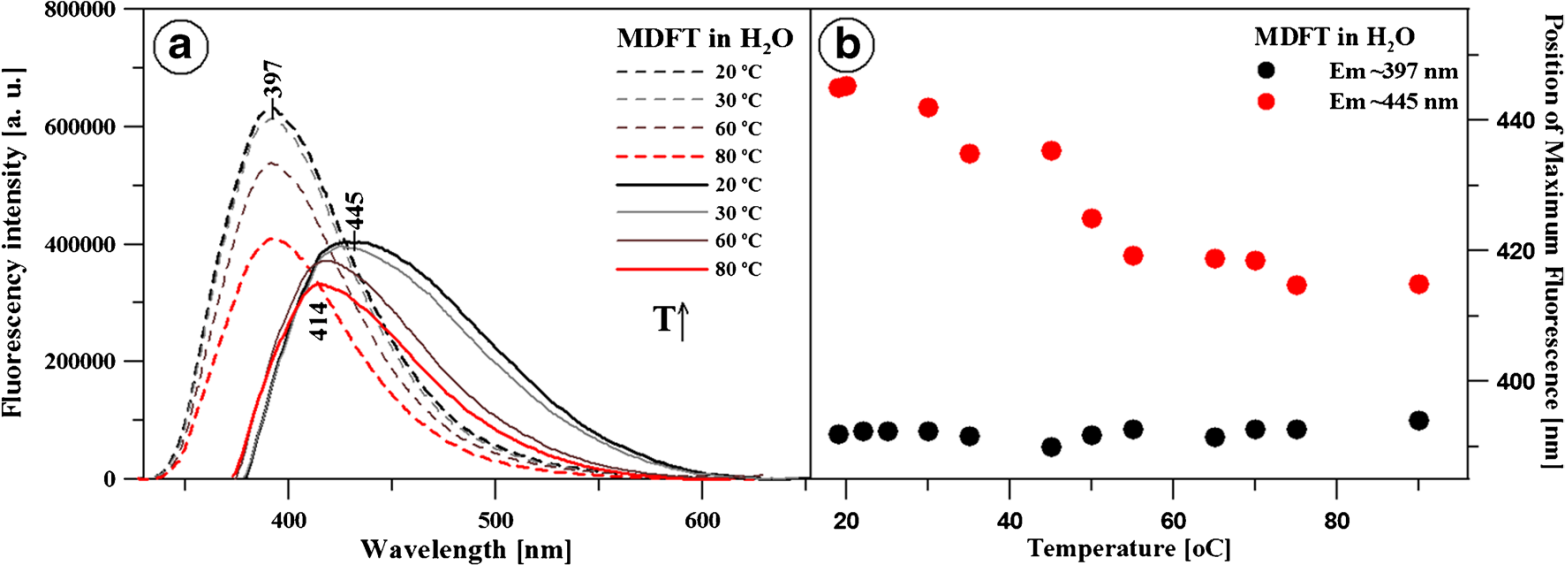
Panel **a** presents the fluorescence emission spectrum of MDFT in the aqueous solution at pH 3 together with the change (rise) in the solution temperature in the range from 20 to 80 °C. The excitation wavelength for the spectra is 316 nm (short-wave emission) and 360 nm (long-wave emission). Panel **b** shows the position of the maxima of the short-wave and long-wave fluorescence emissions together with the rise in the temperature of the aqueous solution from Panel **a**

The next part of the investigations of the fluorescence effects consisted in determination of the effect of temperature on the disaggregation of the analysed systems. Panel a in Fig. 9 presents MDFT fluorescence emission spectra in relation to temperature changes at an excitation wavelength of 397 nm (dashed lines) and 445 nm (solid lines). Evidently, the rise in the temperature is accompanied by a decline in the intensity of both fluorescence maxima. Moreover, along with the temperature rise, there is a very distinct shift of the band characteristic for secondary emission (Em(Ex445)) from 445 to 414 nm. Panel b in this figure shows a change in the position of the two fluorescence maxima (for excitation: to 397 and 445 nm) depending on temperature changes. The panel clearly shows that the position of the first emission maximum does not change with the rise in the environment temperature, but there is a distinct hypsochromic shift of the long wave fluorescence emission maximum. With the temperature decline, the fluorescence maximum is only slightly shifted to the long waves, and the position of primary emission is virtually unchanged (not shown). In order to check whether the compound underwent degradation, NaOH and HCl were added and appropriate fluorescence emission spectra were obtained, which again yielded two separate fluorescence emissions with maxima at 397 and 445 nm (not shown). In the case of the electron absorption spectra for MDFT in water, depending on the temperature fluctuations, only a slight decline in the absorption maximum was observed (not shown). These results clearly demonstrate the impact of the disaggregation process on the presence of the two fluorescence emission forms, which in turn can be associated with the molecular aggregation of the analysed molecules.

### DFT-TDFT Study

Tables 2 and 3 show the calculation results for the two interaction models: the first model assumes a system of monomeric molecules and the other one assumes a system of aggregated dimeric structures. At the assumption of the monomeric system, a single absorption band with at the energy value of 3.981 eV (317 nm) and a single fluorescence band at the energy value of 3.159 eV (393 nm) are predicted. In the dimeric structure model, four states are expected in the interesting range of the absorption spectrum, three of which exhibit substantial intensity. The most important electronic states in terms of comparison with the experimental spectrum include states with vertical transition energies of 3.622 eV (343 nm) and 3.908 eV (312 nm), which practically coincide with the maxima in the experiment, see Figs. 3 and 4. The calculation results indicate agreement of the theoretical results with the experimental data in the case of the fluorescence emission spectra. As shown in Table 2, two fluorescence bands located at the energies of 2.806 eV (442 nm) and 3.307 eV (375 nm) are noted for the dimer system. The slight discrepancy visible in the case of the 375 nm state may result from the substantial width of the experimental spectrum. The DFT/TDDFT calculations reproduce quantitatively (see Tables 2 and 3) the main features of absorption and fluorescence spectra, assuming that the moieties responsible for photochemistry are monomeric MDFT in the higher pH range and protonated aggregates at low pH. Specifically, the calculations for the protonated dimeric form allowed interpretation of the changes in absorption and the appearance of the new fluorescence phenomena in the low energy region of the spectra as resulting from the existence of the new low-energy dimeric excited state.

**Table 2:** Calculated vertical energies of absorption and fluorescence for neutral MDFT monomer and protonated MDFT dimer (DFT/B3LYP in 6-31G** basis set with PCM and LR formalism)

Neutral MDFT monomer				Protonated MDFT dimer			
Absorption							
Excited state	Energy [eV]	Energy [nm]	Osc. Str	Excited state	Energy [eV]	Energy [nm]	Osc. Str
1	3,918	317	0,622	1	3,622	343	0,107
				2	3,789	327	0,019
				3	3,980	312	0,505
				4	4,096	303	0,242
Fluorescence							
Excited state	Energy [eV]	Energy [nm]		Excited state	Energy [eV]	Energy [nm]	
1	3,159	393		1	2,806	442	
				3	3,307	375	

Osc. Str. Oscillator Strength, proportional to intensity

**Table 3:** Experimental absorption and fluorescence energies for neutral and protonated MDFT

	Absorption		Fluorescence	
	Energy [nm]	Energy [eV]	Wavelength [nm]	Energy [eV]
Protonated MDFT	317	3,914	389	3,189
	363	3,418	445	2,788
Neutral MDFT	322	3,853	396	3,133

## Conclusions

The investigations presented in this article and conducted mainly with fluorescence spectroscopy methods clearly indicate emergence of two separate fluorescence bands in the MDFT emission spectrum in an aqueous solution at a pH range from 1 to 3.5. The RLS technique has shown that the observed effect largely depends on chromophore aggregation. Furthermore, the substantial shortening of fluorescence lifetimes evidently confirms the accepted model of MDFT molecular aggregation. It has also been shown that the observed phenomenon can be attributed to the combination of two effects, i.e. specific molecule conformation and aggregation effects, which trigger molecular interactions in the analysed system. This hypothesis is confirmed by the TD-DFT calculations, which yield energy values corresponding to the experimental results obtained for the spectral forms when existence of a protonated dimeric system is assumed. The presented investigations can facilitate rapid fluorescence spectroscopy analysis of structural effects in various biologically important model systems.
